# An Injectable Platform of Engineered Cartilage Gel and Gelatin Methacrylate to Promote Cartilage Regeneration

**DOI:** 10.3389/fbioe.2022.884036

**Published:** 2022-04-14

**Authors:** Wei Xu, Tao Wang, Yahui Wang, Xiaodi Wu, Yujie Chen, Daiying Song, Zheng Ci, Yilin Cao, Yujie Hua, Guangdong Zhou, Yu Liu

**Affiliations:** ^1^ Research Institute of Plastic Surgery, Wei Fang Medical College, Weifang, China; ^2^ National Tissue Engineering Center of China, Shanghai, China; ^3^ Shanghai Key Laboratory of Tissue Engineering, Department of Plastic and Reconstructive Surgery, Shanghai 9th People’s Hospital, Shanghai Stem Cell Institute, Shanghai Jiao Tong University School of Medicine, Shanghai, China; ^4^ Shanghai Engineering Research Center of Nano-Biomaterials and Regenerative Medicine, College of Chemistry, Chemical Engineering and Biotechnology, Donghua University, Shanghai, China; ^5^ Shanghai Resthetic Bio CO., LTD, Shanghai, China

**Keywords:** injectable platform, cell-hydrogel, cartilage regeneration, engineered cartilage gel, immune response

## Abstract

Cell–hydrogel constructs are frequently used as injectable platforms for irregular cartilage regeneration. However, cell–hydrogel constructs have obvious disadvantages, such as long culture times, high probability of infection, and poor cartilage formation capacity, significantly limiting their clinical translation. In this study, we aimed to develop a novel injectable platform comprising engineered cartilage gel (ECG) and gelatin methacrylate (GelMA) to improve cartilage regeneration. We first prepared an ECG by cutting the *in vitro* engineered cartilage sheet into pieces. The chondrocytes and ECG were evenly encapsulated into GelMA to form Cell-GelMA and ECG-GelMA constructs. The ECG-GelMA construct exhibited preferred gel characteristics and superior biocompatibility compared with the Cell-GelMA construct counterpart. After subcutaneous implantation in nude mice and goat, both gross views and histological evaluations showed that the ECG-GelMA construct achieved more homogenous, stable, and mature cartilage regeneration than the Cell-GelMA construct. Immunological evaluations showed that ECG-GelMA had a mitigatory immunologic reaction than the Cell-GelMA construct. Overall, the results suggest that the ECG-GelMA is a promising injectable platform for cartilage regeneration that may advance clinical translation.

## 1 Introduction

Stable cartilage restoration still poses a tremendous clinical challenge for plastic surgeons (Zhou et al., 2018). Developments in tissue engineering technologies have provided a new avenue to achieve cartilage regeneration. When tissue-engineered cartilage is used in plastic surgery, it must create little trauma, have plasticity, be homogeneous, and effectively form cartilage tissue. The emergence of injectable cartilage may solve these problems (Liu et al., 2017).

The basic principle for the development of injectable cartilage is to inoculate chondrocytes into a fluid vehicle (Xu et al., 2020c), inject the chondrocyte-loaded fluid vehicle into the defect to form biologically active cartilage tissue, filling and repairing a defect site (Hua et al., 2021). This method is easily accepted by patients because it is simple, the injectable is malleable, and the process creates little trauma (Cakmak et al., 2013; Singh et al., 2018). Among a variety of fluid vehicles, hydrogels, such as gelatin methacrylate (GelMA), are the most frequently used platform for injectable cartilage tissue engineering because of their remarkable characteristics, including flexibility and versatility in fabrication, variety in composition, high moldability, excellent biocompatibility, and similarity to the extracellular matrix (ECM) (Wu et al., 2020; Xu et al., 2021b). However, because of their compact structure, hydrogels can block nutrient penetration, hindering oxygen and waste exchange, and impeding cell–cell interactions, which significantly interferes with cartilage formation and limits clinical translation (Lee and Shin, 2007; Qi et al., 2015).

Our recent studies have proved that mature *in vitro* engineered cartilage can improve the stability and efficiency of regenerated cartilage *in vivo* (Xu et al., 2020a; Xu et al., 2020d). Consequently, loading hydrogels with more mature cartilage tissue instead of chondrocytes may enhance the quality of the *in vivo* engineered cartilage. Scaffold-free cartilage sheet technology is based on the theory of “chondrocyte redifferentiation in high-density culture” (Schulze-Tanzil et al., 2002; Schuh et al., 2012), and extensive experiments have demonstrated that this technology can regenerate high quality cartilage tissue *in vitro* (Ding et al., 2021). Our latest studies demonstrated that an engineered cartilage gel (ECG) produced by the cartilage sheet technology has the excellent ability to regenerate cartilage (Ci et al., 2021). As a result, we suspect that ECG may be an alternative for chondrocytes, acting as the cellular supplementation in the form of microtissues to load into the GelMA, creating a new injectable platform for cartilage regeneration.

To verify our hypothesis, goat-derived chondrocytes were used to prepare a cartilage sheet, which was further cut into pieces to form the ECG. Thereafter, both chondrocytes and ECG were evenly encapsulated into GelMA to form Cell-GelMA and ECG-GelMA constructs, respectively. We systematically compared the gelation characteristics, biocompatibility, *in vivo* cartilage formation, and inflammatory reaction of the Cell-GelMA and ECG-GelMA constructs, to determine the feasibility of developing a novel injectable platform comprising ECG and GelMA to improve cartilage regeneration.

## 2 Materials and Methods

### 2.1 Chondrocytes Preparation

This study was approved by the Weifang Medical University Ethics Committee. Goats (2,3 months old) were purchased from Shanghai Jiagan Experimental Animal Raising Farm (Shanghai, China). Auricular cartilage obtained from a goat was fragmented into 1 × 1 mm^2^ pieces and digested with 0.2% collagenase NB4 (Worthington biochemical Crop., Freehold, NJ, United States) for 8 h at 37°C on a shaker. The isolated chondrocytes were collected and cultured in Dulbecco’s modified Eagle’s medium (DMEM, Gibco BRL, Grand Island, NY, United States) containing 10% fetal bovine serum (FBS, Gibco BRL) and 1% antibiotic–antimycotic (Gibco BRL). Chondrocytes were passaged at >80% confluence. Chondrocytes at passage 3 (P3) were harvested for use.

### 2.2 ECG Formation

Cartilage sheets were prepared as previously described (Xue et al., 2018; Ci et al., 2021). Briefly, the above prepared chondrocytes were seeded in 6-well cell culture plates at a density of 1.5 × 107 cells/well and cultured in a medium of high DMEM, 10% FBS, and 1% antibiotic–antimycotic. The medium was changed every day, and cartilage sheets were harvested with cell scrapers after 5 days cultivation. ECG was obtained by cutting the obtained cartilage sheet with scissors into small pieces with *an area of 0.1–0.2 mm* ^*2*^.

### 2.3 Preparation of Cell-GelMA and ECG-GelMA Constructs

GelMA was synthesized as previously described (Van Den Bulcke et al., 2000). To prepare the standard hydrogels, 13% GelMA (w/v) was dissolved in phosphate buffered saline (PBS) at 50 °C to create the gel precursors, which were then mixed with 0.2% (w/v) photo-initiator (lithium phenyl-2,4,6-trimethylbenzoyl-phosphinate, LAP). The prepared GelMA solution was filtered aseptically (0.22 µm) and stored at 4°C prior to use.

The harvested chondrocytes were suspended to a concentration of 1.7 × 10^8^ cells/m, and mixed with GelMA at a ratio of 1:1 by volume to achieve a Cell-GelMA construct with a final concentration of 8.5 × 10^7^ cells/ml.

The collected ECG was mixed with GelMA at a ratio of 1:1 by volume, achieving a ECG-GelMA construct with a final concentration of 8.5 × 10^7^ cells/mL. Both the Cell-GelMA and ECG-GelMA constructs were crosslinked by UV light irradiation (365 nm, 20 mW/cm^2^) for 5 s.

### 2.4 Characteristics of the GelMA, Cell-GelMA, and ECG-GelMA Constructs

#### 2.4.1 Morphology

The chondrocytes, cartilage sheet, ECG, GelMA, Cell-GelMA, and ECG-GelMA were observed using a light microscope (OLYMPUS, CKX41), and photographed using a single lens reflex camera (Nikon, Japan). The GelMA, Cell-GelMA, and ECG-GelMA constructs were freeze-dried and examined using scanning electron microscopy (SEM, Philips XL-30, Amsterdam, Netherlands) at an accelerating voltage of 15 kV.

#### 2.4.2 *Rheological Analyses and Viscosity*

Tests of the viscosity and shear-thinning behavior of the gel precursors were performed on a HAAKE MARS Rotational Rheometer with a parallel plate geometry (P20 TiL, 20 mm diameter) at 25°C. The shear-thinning behavior was analyzed at a 0.5 mm gap from 0 1/s to 50 1/s. Dynamic rheology experiments were exposed to light irradiation (365 nm, 20 mW/cm^2^) within 60 s. A time sweep oscillatory test was performed at 10% strain (CD mode), 1 Hz frequency, and a 0.5 mm gap for 120 s. The gel point was determined as the time when the storage modulus (G′) surpassed the loss modulus (G″).

#### 2.4.3 Swelling Ratio and In Vitro Enzymatic Degradation

The initial volume of the GelMA, Cell-GelMA, and ECG-GelMA constructs was recorded as V_0_, and the volume after 24 h was recorded as V. The volume swelling ratio was defined as SRv = V/V_0_ (Gan et al., 2019).

$$SR_{V}=\frac{\text{π}R_{1}^{2}\times H_{1}}{\text{π}R_{0}^{2}\times H_{0}}$$

R_1_ and R_0_ are the radii of the various hydrogels before and after 24 h swelling, respectively, and H_0_ and H_1_ are the initial height and the height after 24 h swelling of the various hydrogels, respectively.

The enzymatic degradation was examined by a gravimetric method, as previously described (Xu et al., 2020d). The initial hydrogel dry weight was W_0_ and the remaining hydrogel dry weight after immersing in an enzymatic solution (0.15% w/v collagenase) was recorded as W_t_. The degradation ratio was defined as W_t_/W_0_×100%.

#### 2.4.4 Mechanical Testing

The mechanical properties of the hydrogel discs (Φ 10 × 3 mm), including GelMA, Cell-GelMA, and ECG-GelMA constructs, were examined using a mechanical testing machine (Instron-5542, Instron, Canton, MA, United States), as previously described (Gan et al., 2019). Briefly, crosslinked samples were compressed at a rate of 0.5 mm/min at room temperature until 80% of the maximum deformation to obtain compressive stress–strain curves. The compressive modulus was then calculated from the slope of the stress–strain curve.

#### 2.4.5 Biocompatibility of the Cell-GelMA and ECG-GelMA Constructs

To determine the biocompatibility of the Cell-GelMA and ECG-GelMA constructs, chondrocytes within the constructs were adjusted to a final concentration of 3.0 × 10^7^ cells/mL. After 1, 4, and 7 days *in vitro* culture, the viability of the cells within the constructs were evaluated using a Live and Dead Cell Viability Assay (Invitrogen, United States) *via* a confocal microscope (Nikon, Japan), following the manufacturer’s instructions. After imaging using the confocal microscope, ImageJ software was used to quantitatively analyze the number of dead cells.

To observe the chondrocytes and ECG morphology within GelMA, samples after 7 days *in vitro* culture were washed thrice in PBS and the samples were fixed in 4% paraformaldehyde (PFA) at room temperature for 30 min. The fixed samples were washed thrice in PBS and immersed in a permeabilization and blocking buffer (1% bovine serum albumin and 0.1% Triton in PBS) for 30 min. To stain the actin cytoskeleton, a diluted methanolic solution of Alexa Fluor 546 phalloidin (Invitrogen) in the blocking buffer was incubated with the fixed samples at room temperature in the dark for 30 min. All samples were washed with PBS and the nuclei was counterstained with 4, 6-diamidino-2-phenylindole dihydrochloride (DAPI). All samples were sealed with a solution containing an anti-fluorescence quenching agent. The images were acquired using a confocal microscope equipped with the appropriate excitation and emission filters.

The DNA content was extracted from the samples using 250 μg/ml Protease K, and then quantified with the PicoGreen dsDNA assay (Invitrogen) according to the manufacturer’s protocol to determine the chondrocytes proliferation within the constructs.

### 2.6 Subcutaneous Injection Into Nude Mice and Goats

Cell-GelMA and ECG-GelMA constructs were subcutaneously injected into the dorsal area of nude mice at a total volume of 0.15 ml via a syringe with an 18-gauge needle. Samples were harvested 8 weeks post-implantation. Cell-GelMA, ECG, and ECG-GelMA constructs were also subcutaneously injected into the dorsal area of goats at a total volume of 0.4 ml. Samples were harvested at 1, 2, and 12 weeks post-implantation.

### 2.7 Quantitative Polymerase Chain Reaction (qPCR)

The expression of cartilage-related genes (ACAN, COL-2, and Sox-9) and inflammation-related genes (CD3 and CD68) were analyzed by qPCR. The total RNA was extracted with Tiazol reagent (Invitrogen), and then reverse transcribed using Moloney murine leukemia virus reverse transcriptase (Invitrogen). qPCR was performed using a Fast Synergy Brands Green Master Kit and Light Cycler 480 system (Roche) in accordance with the manufacturer’s instructions. The results were analyzed using the comparative threshold cycle method and normalized to endogenous reference gene GAPDH. The primer sequences are listed in Table 1.

**TABLE 1:** Primer sequences of related genes.

Gene	Accession number	Primer sequence
ACAN	XM_018066613.1	CAG​AGG​CAA​CCA​CAA​CAG​ACA
		AGC​TGG​GAA​GGC​ATA​AGC​ATG
COL-2	XM_018047868.1	GCA​TTG​CCT​ACC​TGG​ACG​AAG TCA​CAG​TCT​CGC​CCC​ACT​TAC
Sox-9	XM_018063905.1	AAG​AAC​AAG​CCG​CAC​GTC​AA CCG​TTC​TTC​ACC​GAC​TTC​CTC
CD3	XM_005689508.3	TTA​TCA​GTG​CCT​CGC​AAC​CG CTT​TCG​GCT​CTT​GCT​CCA​GTA
CD68	XM_005693517.3	AGC​CCA​GAT​TCA​GAT​GCG​AGT GAT​CCT​GTT​TGA​ATC​CGA​AGC​T

### 2.8 Histological and Immunohistochemical Analyses

Samples were fixed in 4% paraformaldehyde and embedded in paraffin for sectioning into 5-µm thick slices that were then mounted on glass slides. Sections were stained with hematoxylin and eosin (HE) and safranin-O (SO). For immunohistochemical analysis, a rabbit anti-human monoclonal antibody against collagen II (COL-2) was used with a horseradish peroxidase (HRP)-conjugated anti-rabbit antibody (1:400 in PBS, Santa Cruz) as the secondary antibody. CD3 was detected using a rabbit anti-human CD3 monoclonal antibody (1:100 in PBS, Santa Cruz Biotechnology, Santa Cruz, CA, United States). CD68 was detected using a rabbit anti-human CD68 monoclonal antibody (1:1,000 in PBS, Santa Cruz Biotechnology). Color development was conducted with diaminobenzidine tetrahydrochloride (Santa Cruz Biotechnology).

### 2.9 Biochemical and Biomechanical Determination

Quantitative biochemical analysis of regenerated cartilage was performed as described previously (Wang et al., 2020; Chen et al., 2021). Briefly, samples were weighed using an electronic balance. The volume of each sample was measured using a water displacement method. The GAG content in the samples was quantified using Alcian Blue. The total collagen content was detected using a hydroxyproline assay. The DNA content was determined as described above. *The amount of COL-1 and COL-2 was measured by an ELISA.*

Young’s modulus was analyzed using a constant compressive strain rate in a biomechanical analyzer (Instron-5969, Canton, MA, United States), as previously described (Xu et al., 2020b). The Young’s modulus was calculated using the slope of the stress–strain curve.

### 2.10 Statistical Analysis

All samples were test at least three times. Differences in quantitative data were analyzed using SPSS23. A *p* value < 0.05 was considered statistically significant.

## 3 Results

### 3.1 Characterizations of ECG, Cell-GelMA, and ECG-GelMA Constructs

**FIGURE 1:**
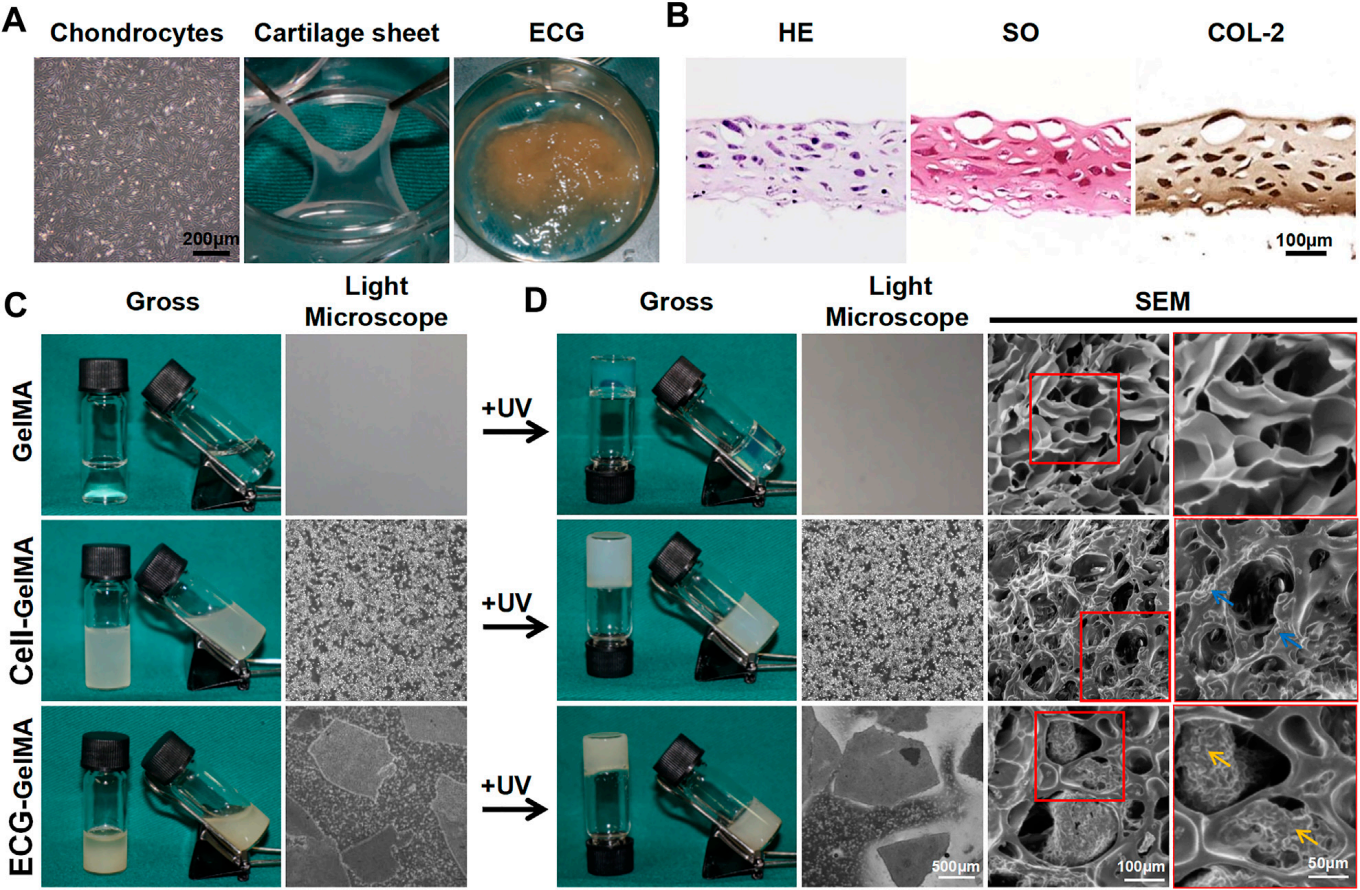
The synthesis and gel properties of ECG-GelMA. The light microscope image of chondrocytes at P3, gross image of cartilage sheet after 5 days *in vitro* cultured, and gross image of ECG derived from chopped cartilage sheet **(A)**. HE, SO, and COL-2 staining of cartilage sheet **(B)**. The gross and light microscope images of GelMA, Cell-GelMA, and ECG-GelMA before UV crosslinking **(C)**. The gross, light microscope, and SEM images of GelMA, Cell-GelMA, and ECG-GelMA after UV crosslinking **(D)**. The blue arrows indicate cells, and the orange arrows indicate ECG.

As shown in the light microscopy images in Figure 1A, P3 chondrocytes had polygonal morphology, indicating viability. The chondrocytes were cultured in 6-well culture plates at a density of 1.5 × 10^7^ cells/well for 5 days, successfully achieving a cartilage sheet with pink-white appearance and soft texture. The generated cartilage sheet had lacunae structures and preliminary cartilage-specific ECM deposition, as confirmed by superficial SEM examination (Supplementary Figure S1A), as well as positive SO and COL-2 staining (Figure 1B). Phalloidine staining further revealed that the cartilage sheet displayed extensive F-actin (Supplementary Figure S1B), indicating favorable viability.

Thereafter, the generated cartilage sheet was cut into pieces to achieve ECG, which had satisfactory gel properties and could be collected using a 5 ml syringe (Supplementary Figure S2 and Supplementary Videos S1). The P3 chondrocytes and ECG were evenly blended with GelMA to form Cell-GelMA and ECG-GelMA constructs, respectively. Our results confirmed that both the Cell-GelMA and ECG-GelMA constructs exhibited gel properties (Supplementary Videos S2,S3).

As shown in the gross view and light microscope images (Figure 1C), a uniform distribution of chondrocytes and ECG were clearly observed inside the GelMA, and the same results were observed after being exposed to UV light irradiation (Figure 1D). SEM images revealed that the freeze-dried pure GelMA exhibited highly interconnected micropores with smooth pore walls, whereas chondrocytes aggregated on the pore walls in the Cell-GelMA group and minced ECG was distributed in the pores in the ECG-GelMA group.

**FIGURE 2:**
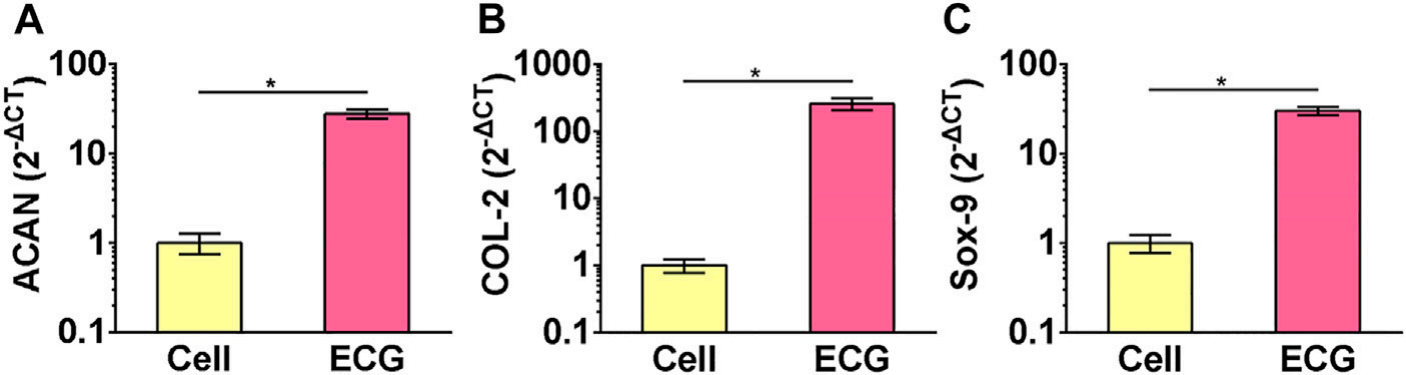
QPCR analysis of chondrocytes and ECG. Gene expression of ACAN **(A)**, COL-2 **(B)** and Sox-9 **(C)** in chondrocytes and ECG after 5 days *in vitro* culture. *p < 0.05.

The gene expression of the chondrocyte and ECG groups were examined using qPCR. The expression of cartilage-related genes, including ACAN, COL-2, and Sox-9, was significantly greater in the ECG than in chondrocytes after 5 days *in vitro* culture (Figures 2A–C).

The Cell-GelMA and ECG-GelMA constructs exhibited injectable gel modality with suitable viscosity, and the addition of ECG into GelMA enhanced the viscosity of the hydrogel (Supplementary Figure S3A). Time-sweep rheological analyses further confirmed fast gelation at approximately 5 s, indicating satisfactory photo-crosslinked performance of gel precursors (Supplementary Figure S3B). Adding ECG to the GelMA hydrogel significantly enhanced the storage moduli (Supplementary Figure S3C), whereas it did not affect the compressive moduli (Supplementary Figure S3D). The GelMA, Cell-GelMA, and ECG-GelMA groups displayed a consistent low swelling ratio (Supplementary Figure S3E), which is beneficial for *in vivo* implantation and shape maintenance. *In vitro* degradation experiments showed that all samples significantly degraded over the initial 8 h (Supplementary Figure S3F), suggesting favorable biodegradability for cartilage regeneration. Furthermore, our results confirmed that both the Cell-GelMA and ECG-GelMA constructs were quickly crosslinked from the solution to the sol state upon light irradiation (365 nm, 20 mW/cm^2^) within 60 s (Supplementary Figure S4). Alternatively, pure ECG failed to form a gel state under identical light irradiation. Collectively, the results indicate the successful preparation of Cell-GelMA and ECG-GelMA constructs that may be ideal injectable gel modalities for cartilage regeneration.

### 3.2 Biocompatibility

**FIGURE 3:**
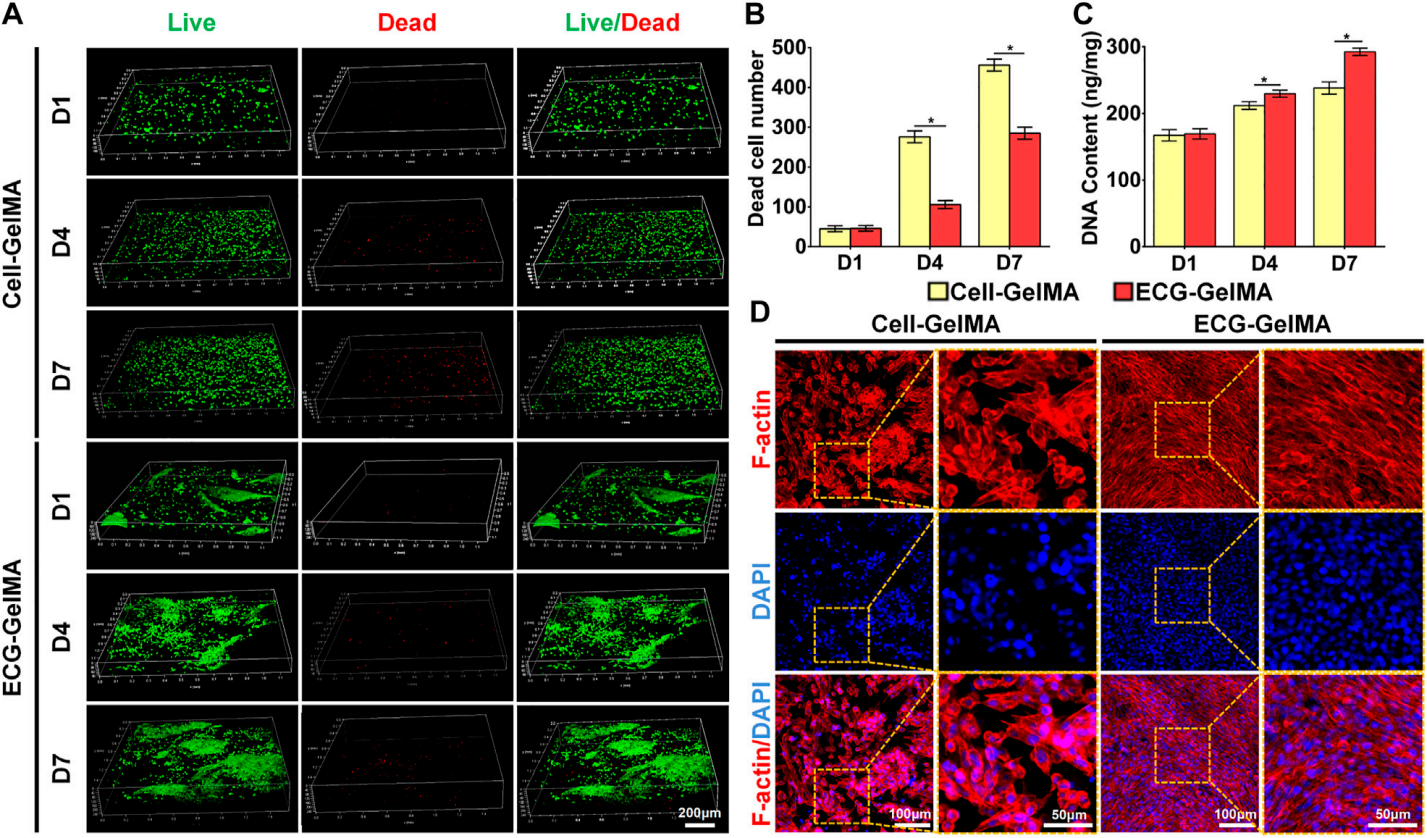
Biocompatibility evaluation of Cell-GelMA and ECG-GelMA. Live/dead staining images **(A)**, as well as quantitative analysis of dead cell number **(B)** and DNA content **(C)** in Cell-GelMA and ECG-GelMA groups at 1–7 days. Phalloidin staining images in Cell-GelMA and ECG-GelMA groups at 7 days **(D)**. *p < 0.05.

Cell viability assays showed that chondrocytes grew well in both Cell-GelMA and ECG-GelMA with significant proliferation rates and few dead cells at 1, 4, and 7 days (Figure 3A). Notably, significantly more dead cells were observed in the Cell-GelMA group, whereas few dead cells were displayed in the ECG-GelMA group, as further confirmed by quantitative analysis of the dead cells (Figure 3B). In addition, the cell proliferation test demonstrated that the total DNA content in both the Cell-GelMA and ECG-GelMA groups gradually increased with increasing culture time, and more DNA was observed in the ECG-GelMA group than in the Cell-GelMA group (Figure 3C). Phalloidine staining showed that the ECG-GelMA construct displayed extensively more F-actin and stretched more than the Cell-GelMA construct (Figure 3D). Collectively, these results indicate that ECG had better biocompatibility than the cells (chondrocytes) when encapsulated within GelMA.

### 3.3 Cartilage Regeneration in a Nude Mice Model

**FIGURE 4:**
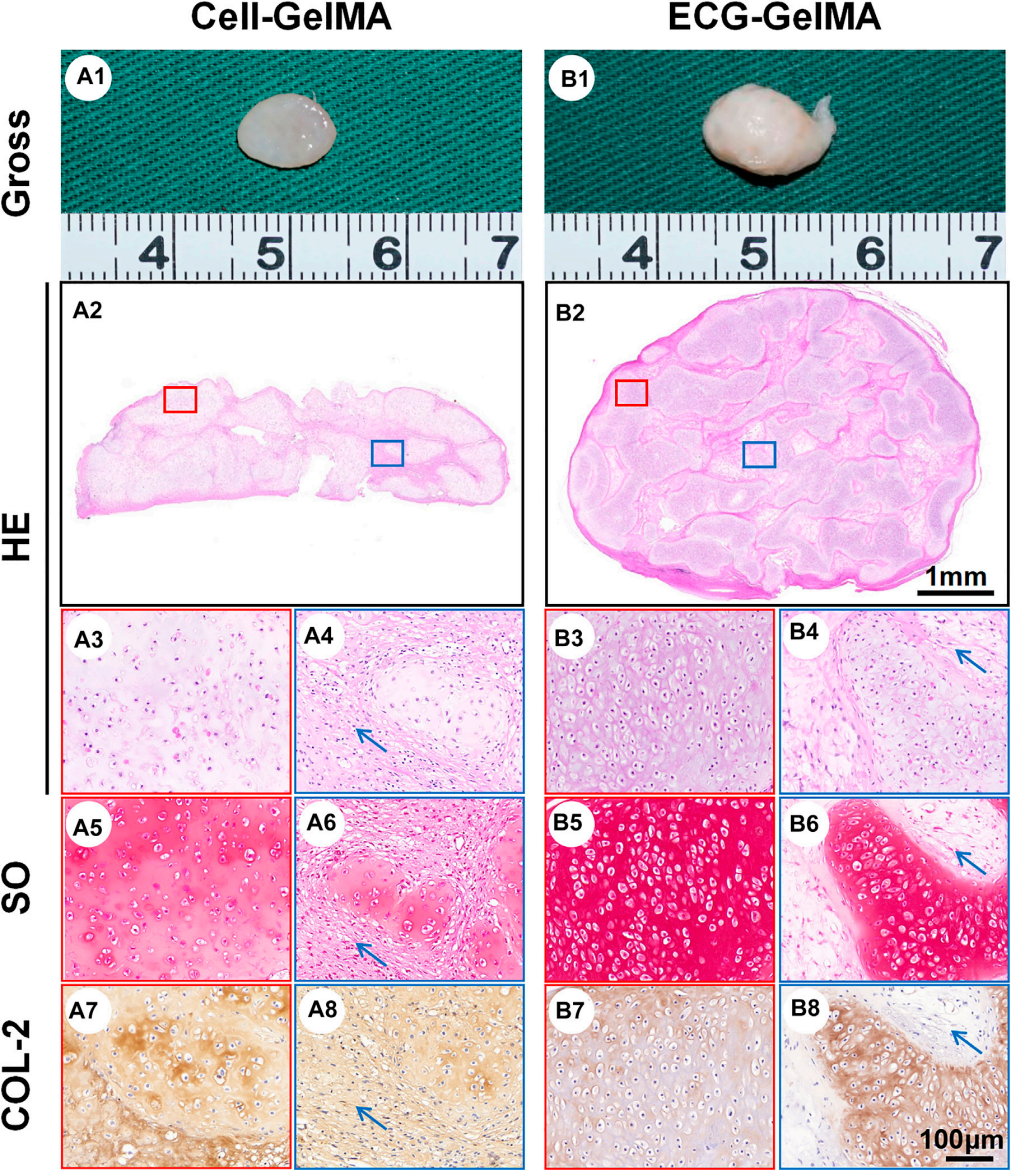
Gross view and histological examination of regenerated cartilage in Cell-GelMA and ECG-GelMA after 8 weeks implantation in nude mice. Gross view (A1 and B1), HE (A2-A4 and B2-B4), SO (A5-A6 and B5-B6), and immunohistochemical COL-2 (A7-A8 and B7-B8) staining in Cell-GelMA and ECG-GelMA groups. The blue box represents magnified images at inner zone of regenerated cartilage, and the red box represents magnified images at peripheral zone of regenerated cartilage. The blue arrows indicate fibrous tissue.

**FIGURE 5:**
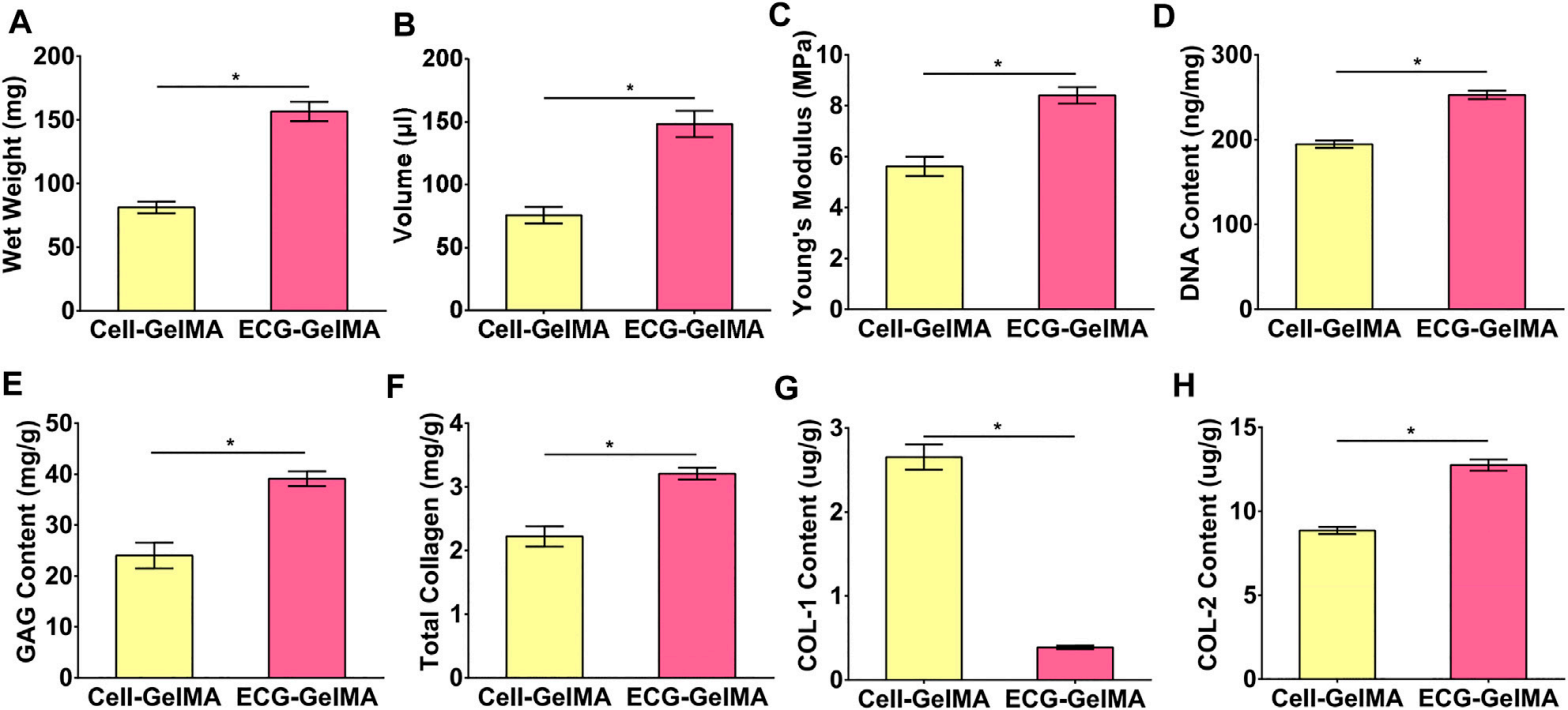
Quantitative analysis of the regenerated cartilage in Cell-GelMA and ECG-GelMA groups after 8 weeks implantation in nude mice. Quantitative analysis of wet weight **(A)**, volume **(B)**, Young’s modulus **(C)**, DNA content **(D)**, GAG content **(E)**, total collagen **(F)**, COL-1 content **(G)**, and COL-2 content **(H)** in Cell-GelMA and ECG-GelMA groups. *p < 0.05.

The feasibility of cartilage regeneration was primary tested in nude mice. After 8 weeks subcutaneous implantation, both Cell-GelMA and ECG-GelMA successfully generated cartilage-like tissues with an ivory-white appearance (Figure 4A1 and B1). Histological analysis further confirmed that samples in all groups formed cartilage-specific ECM distributions with abundant lacuna structures (Figure 4A2-A8 and B2-B8). Notably, the ECG-GelMA group exhibited a significantly robust appearance with larger volume and homogeneous cartilage ECM deposition than the Cell-GelMA group (Supplementary Figure S5). Additionally, heterogeneous cartilage ECM distribution and fibrous-like tissue were observed in Cell-GelMA (Figure 4A2), especially in the inner zone (Figure 4A4, A6, and A8). Consistent with the gross and histological staining, the quantitative analysis showed that the wet weight, volume, Young’s modulus, DNA content, GAG content, total collagen content and *COL-2 content* presented a trend of ECG-GelMA > Cell-GelMA (Figures 5A–G). COL-1 content showed ECG-GelMA group were less than Cell-GelMA group (Figure 5H). The data suggests that ECG-GelMA outperformed the Cell-GelMA group in terms of cartilage regeneration in nude mice.

### 3.4 Immune Response

**FIGURE 6:**
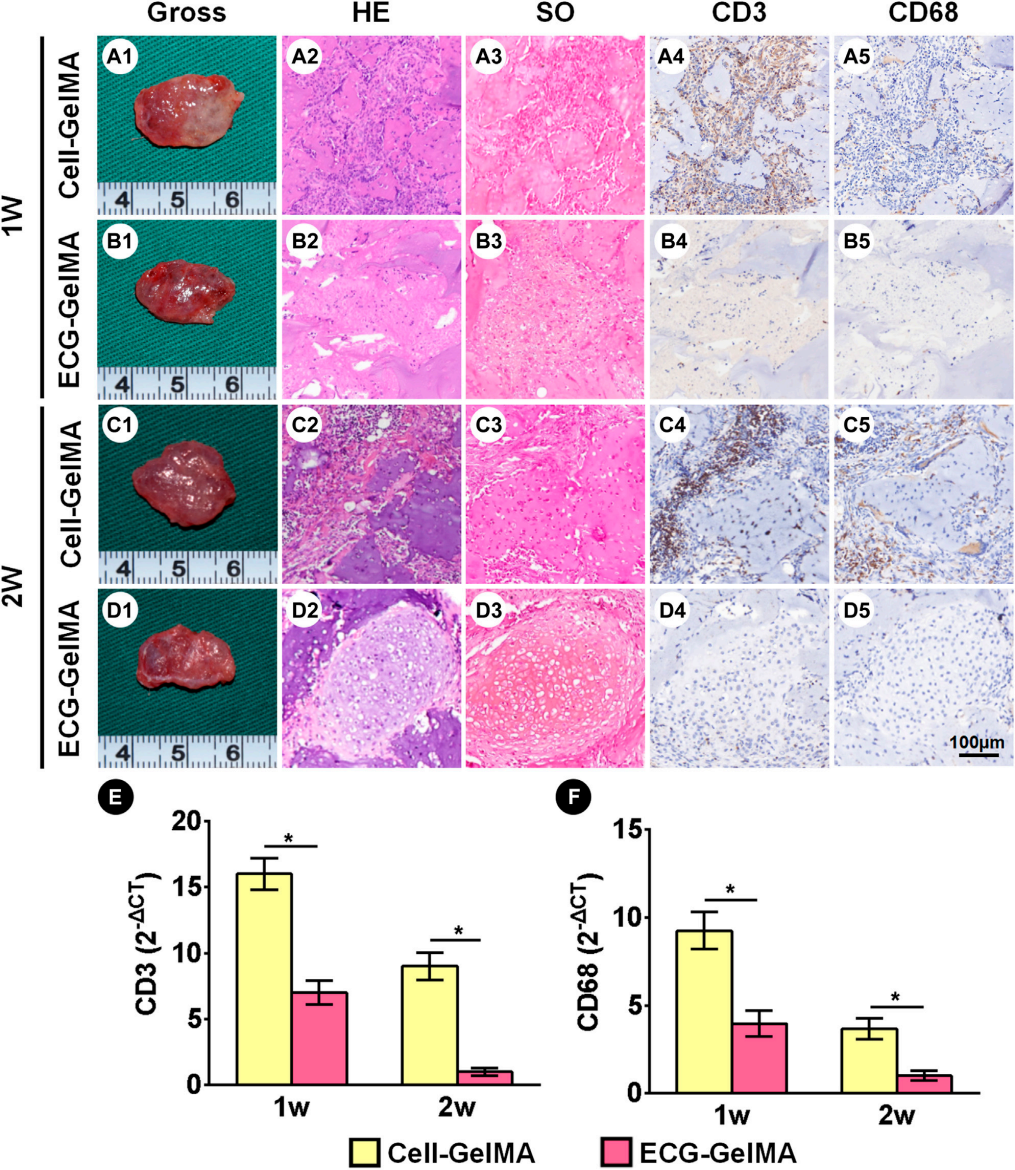
Inflammatory response evaluations of Cell-GelMA and ECG-GelMA constructs subcutaneously implanted into goats for 1 and 2 weeks. Gross view of samples in Cell-GelMA, ECG, and ECG-GelMA groups subcutaneously implanted into goats for 1 week (A1-B1) and 2 weeks (C1-D1). HE (A2-D2), SO (A3-D3), immunohistochemical CD3 (A4-D4) and CD68 (A5-D5) staining of samples in Cell-GelMA, ECG, and ECG-GelMA groups subcutaneously implanted into goats for 1 and 2 weeks. Expression of CD3 (E) and CD68 (F) genes Cell-GelMA, ECG, and ECG-GelMA groups subcutaneously implanted into goats for 1 and 2 weeks *p < 0.05.

The immunological response of the Cell-GelMA and ECG-GelMA groups were compared in an immunocompetent goat model. After 1 week of subcutaneous implantation, samples in the Cell-GelMA group showed tremendous inflammatory cell infiltration, indicated by positive immunohistochemical staining for CD3 and CD68 (Figure 6A1-A5). In contrast, little inflammatory cell infiltration was observed in ECG-GelMA (Figure 6B1-B5). Although the inflammatory reaction was significantly reduced in both groups after 2 weeks implantation (Figure 6C1-C5 and D1-D5), obvious inflammatory cells were observed in samples at the Cell-GelMA group, whereas they nearly disappeared in the ECG-GelMA group. qPCR analysis of the inflammation-associated genes was used to evaluate the immune response to xenogeneic tissue (Figures 6E,F). Expression levels of CD3 and CD68 in the Cell-GelMA was much greater than those in the ECG-GelMA after 1 and 2 weeks *in vivo*. The expression levels of CD3 and CD68 in both samples gradually decreased over the *in vivo* period and were nearly undetectable in the ECG-GelMA group after 2 weeks implantation. The ECG-GelMA had negligible inflammatory reaction compared with the Cell-GelMA group.

### 3.5 Cartilage Regeneration in a Goat Model

**FIGURE 7:**
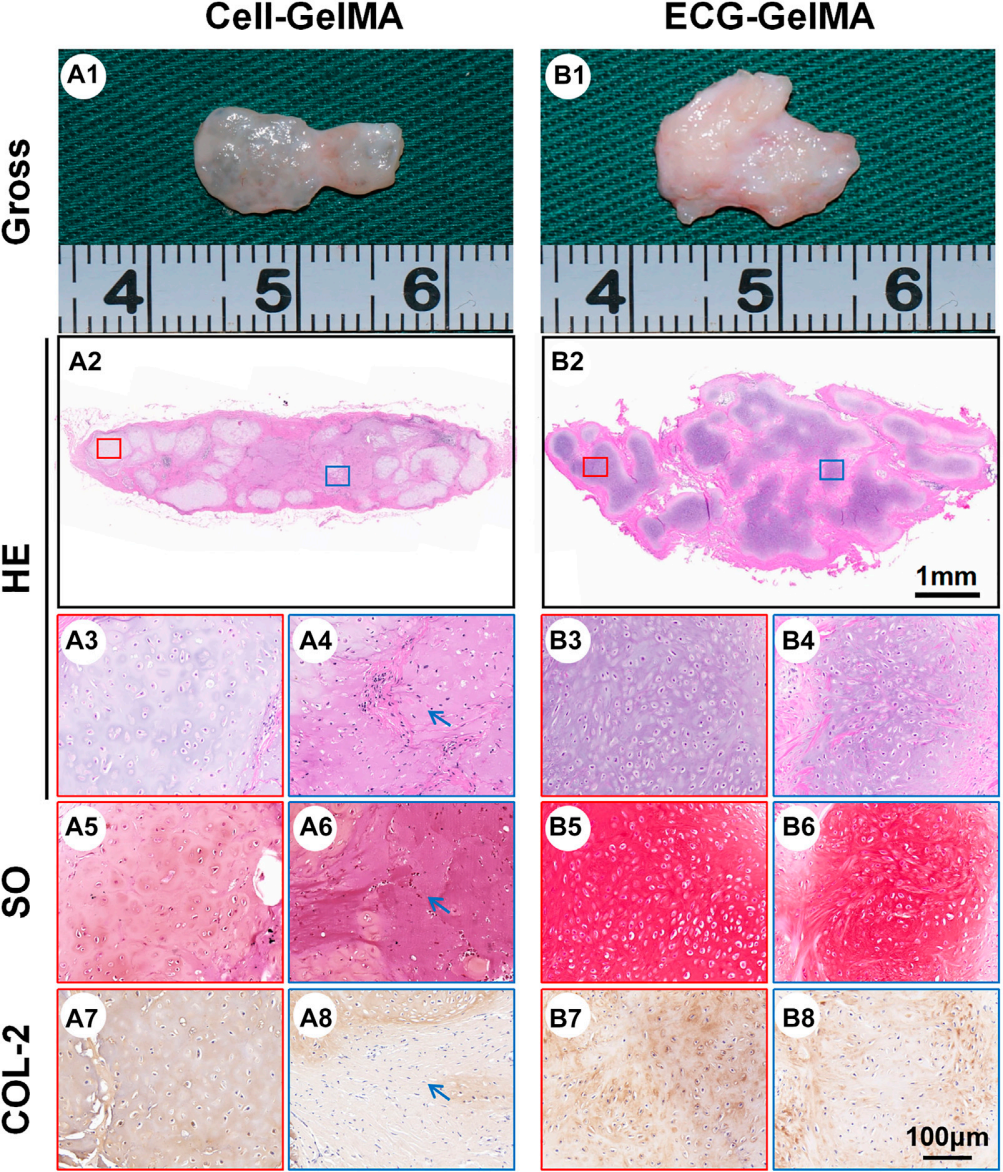
Cartilage regeneration of Cell-GelMA and ECG-GelMA subcutaneously implanted in goat for 12 weeks. Gross view (A1 and B1), HE (A2-A4, and B2-B4), SO (A5-A6 and B5-B6), and immunohistochemical COL-2 (A7-A8 and B7-B8) staining of Cell-GelMA **(A)**, and ECG-GelMA **(B)**. The blue box represents magnified images at inner zone of regenerated cartilage, and the red box represents magnified images at peripheral zone of regenerated cartilage. The blue arrows indicate fibrous tissue.

**FIGURE 8:**
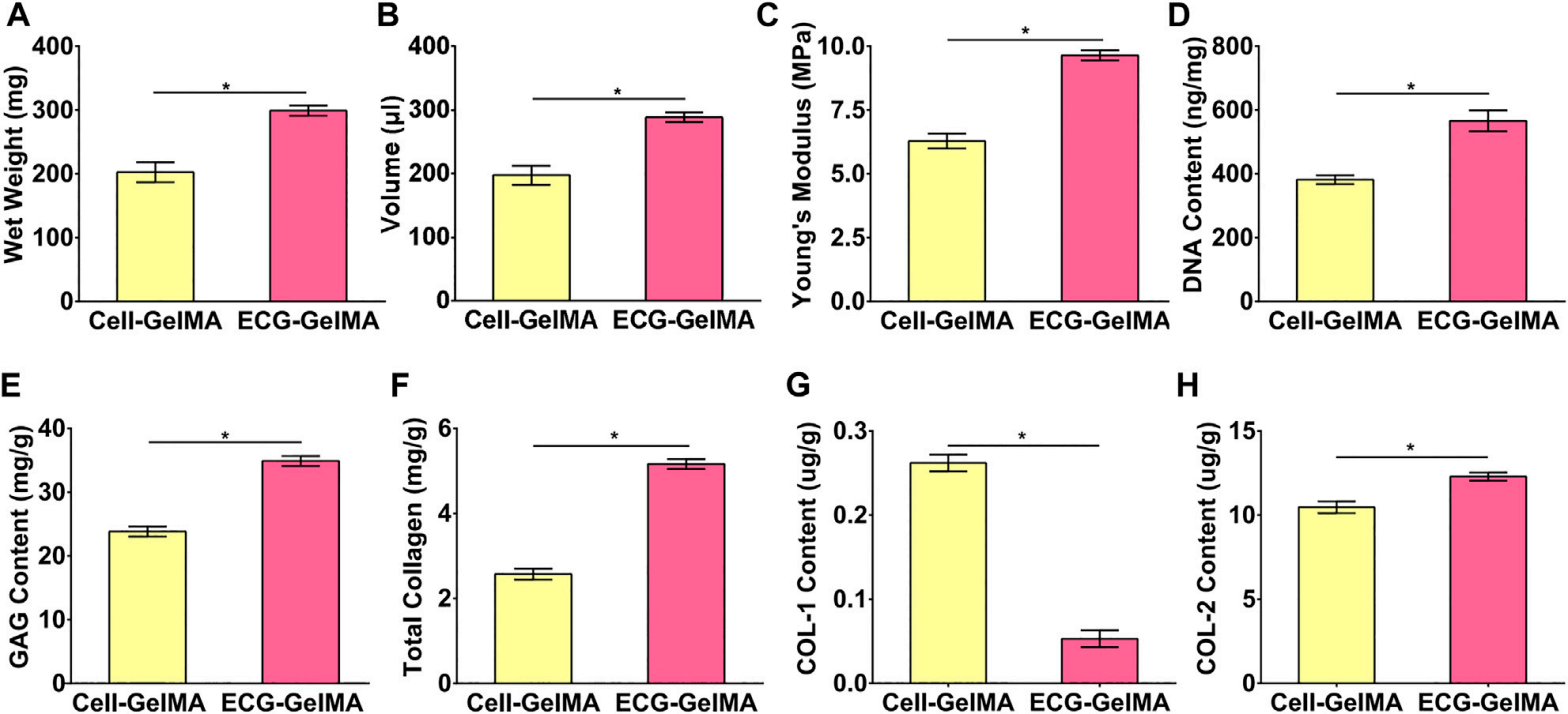
Quantitative analysis of the regenerated cartilage in Cell-GelMA and ECG-GelMA groups after 12 weeks subcutaneously implantation in a goat. Quantitative analysis of wet weight **(A)**, volume **(B)**, Young’s modulus **(C)** DNA content **(D)**, GAG content **(E)**, total collagen **(F)**, COL-1 content **(G)**, and COL-2 content **(H)** in Cell-GelMA and ECG-GelMA groups. *p < 0.05.

The cartilage regeneration abilities of Cell-GelMA and ECG-GelMA were evaluated by subcutaneously injecting the samples into autologous goats. As the *in vivo* period increased, Cell-GelMA and ECG-GelMA gradually matured, as evidenced by the switch from a reddish appearance and less ECM deposition at 1 and 2 weeks to an ivory-white appearance and mature ECM deposition at 12 weeks (Figures 6, 7). Notably, gross and panorama HE staining revealed that the Cell-GelMA construct shrunk during the *in vivo* period, whereas ECG-GelMA showed limited shrinkage (Figure 7A1, B1, A2, B2). Histological results further demonstrated that the regenerated tissues gradually matured over 12 weeks implantation, and GelMA in all groups degraded over time (Supplementary Figure S6). Additionally, immature cartilage-like tissue and undegraded GelMA were observed inside the Cell-GelMA (Figure 7A2-A8), whereas mature and homogeneously distributed cartilage-like tissue was observed in the ECG-GelMA group (Figure 7B2-B8). The biomechanical and biochemical results further indicated that *the wet weight, volume, Young’s modulus, DNA content, GAG content, total collagen and COL-2 content presented a trend of ECG-GelMA > Cell-GelMA* (Figure 8A-G), meanwhile, COL-1 content showed ECG-GelMA group were less than Cell-GelMA group *(* Figure 8H *)*, indicating the advantages of ECG-GelMA for cartilage regeneration in an immunocompetent goat.

## 4 Discussion

Various types of irregular cartilage tissue deficiencies are common clinically, especially in plastic surgery, such as head and face cartilage defects or dysplasia (Pascual-Garrido et al., 2018). Autologous bone, cartilage, and various artificial materials are frequently used to repair cartilage defects or simply improve the morphology by transplantation or injection filling; however, these methods have not been widely used because of their limitations (Schuurman et al., 2013; Wei et al., 2021). Herein, we introduce a new injectable platform for cartilage regeneration that includes cutting an *in vitro* engineered cartilage sheet into pieces to achieve ECG and encapsulating the ECG into GelMA to form an injectable ECG-GelMA construct. Thereafter, the ECG-GelMA construct could be injected *in situ* for *in vivo* cartilage restoration. Our results confirmed that the ECG-GelMA construct supported better cartilage regeneration than a traditional Cell-GelMA construct.

In recent years, injectable cartilage has been easily accepted by patients and has broad application prospects because of unique advantages such as minimal trauma, injection moldability, and convenient operation. Cell–hydrogel constructs are frequently used in the research on injectable cartilage. However, cell–hydrogel constructs still have obvious disadvantages such as long culture times, high probability of infection, and poor cartilage formation capacity (Bryant et al., 2007; Mashingaidze et al., 2013; Ni et al., 2014).

A cartilage sheet is an ideal tissue for tissue-engineered cartilage regeneration, and a photocrosslinkable hydrogel is a promising scaffold for cartilage regeneration. However, using ECG in combination with photo-crosslinked hydrogels *in vitro* to construct injectable cartilage patterns has not been demonstrated. Our current study showed that the ECG-GelMA constructs supported satisfactory chondrogenesis, and was used as an injectable hydrogel for cartilage regeneration.

ECG-GelMA regenerated better quality cartilage with more efficiency than traditional a Cell-GelMA. The ECG-GelMA group had more mature chondrocyte formation than the Cell-GelMA group after 8 weeks implantation in nude mice and 12 weeks in goats. We suggested that the ECG-GelMA is a promising method for cartilage regeneration. The regeneration process and principle are mainly as follows: 1) the ECG was made of tissue pieces from a cartilage sheet cultured *in vitro*, in which genes (ACAN, COL-2, Sox-9) were up-regulated and ECM was preliminary deposited. In the active stage of cartilage regeneration, the implantation can adapt to the internal environment more quickly *in vivo*, and cartilage regeneration is more stable; 2) ECG contains cartilage-specific ECM, allowing chondrocytes to be housed in an environment that mimics their natural growth process and cells may sense the resistance of matrix through attachment to their own ECM, in order to appropriately induce cellular differentiation and tissue remodeling. (Schulze-Tanzil et al., 2002; Xu et al., 2021a); 3) cells are effectively “caged” away from one another by the hydrogel (Zhang et al., 2016), resulting in interference with the fluidity and interaction of chondrocytes in the Cell-GelMA group. In contrast, ECG is a microscale tissue with integrated structure that does not affect cell interaction and is more conducive to cartilage tissue regeneration. 4) Our results confirmed that ECG-GelMA group exhibited mitigative inflammatory reaction compared to Cell-GelMA group, which was obvious conducive for cartilage formation.

The stability of injectable cartilage regeneration in immunocompetent large animals is still a bottleneck because the scaffold materials cause inflammation, which hinders cartilage regeneration (Luo et al., 2009). Prolonged *in vitro* cultivation supports the formation of a mature cartilaginous graft to resist the acute host response and promotes stable subcutaneous cartilage formation in autologous immunocompetent animals (Liu et al., 2016). However, a long-term *in vitro* cultured cell–hydrogel construct still cannot achieve satisfactory cartilage regeneration *in vivo*, mainly because the scaffold degradation is incomplete. The cartilage sheet can improve cartilage regeneration *in vivo* (Wu et al., 2020). However, our previous study found that ECG had a certain degree of absorption in large animals, which may be related to the slight inflammation caused by surgical trauma during the early stages of implantation. A scaffold that can protect ECG from inflammatory cells in the early stages *in vivo* is needed. Herein, we confirmed that the incorporation of GelMA allowed the construct to serve as an immune barrier against the immune response during the initial implantation.

*ECG has unique advantages in clinical translation because of its scaffold-free nature; however, whether the ECG can effectively avoid the inflammatory reaction and steadily maintain the cartilage phenotype in large and immunocompetent animals still needs to be investigated (* *Wu et al., 2020* *). We speculate that GelMA may protect chondrocytes from inflammatory cells. The reasons are as follows. 1) Inflammatory cells were mainly distributed in the periphery of the tissue and very few entered the tissue. 2) A significantly reduced inflammatory response was observed in both of the groups at 2 weeks. In addition, our study demonstrated that the ECG-GelMA group had low inflammatory response and a high cartilage survival rate. We suppose the main reasons are as follows: 1) Numerous studies have confirmed that chondrocytes caused inflammatory responses, while cartilage-specific ECM was inflammatory inert. Compared to the chondrocytes in Cell-GelMA group, chondrocytes in ECG-GelMA group were enveloped by a layer of cartilage-specific ECM in ECG, thus protecting chondrocytes from inflammatory invasion (* *Yang et al., 2005*; *Wu et al., 2007*; *Weidenbecher et al., 2008* *). 2) qPCR analysis demonstrated that the expression of cartilage-related genes, including ACAN, COL-2, and Sox-9 of the ECG was significantly up-regulated, which also promoted the deposition of ECM. The ECM surrounding the cells in ECG function as a natural barrier to make them less vulnerable to inflammatory cells (* *Xu et al., 2019* *). 3) The ECM in ECG-GelMA also provides a suitable microenvironment for cells to reside, and would be more conducive to keep stable cartilage phenotype and lower inflammatory response after implantation compared with Cell-GelMA.*

Although cartilage can be regenerated in immunocompetent animals, how to solve the problem of early inflammation, and how to adjust the ECG construction and hydrogel selection need to be investigated. Furthermore, the generated cartilage was nearly an island structure, which may relate to the ingrowth of connective tissue after injection into the body. The island cartilage is an absorption risk that needs to be solved. Although ECG-GelMA at a 1:1 ratio is reported in this study, whether a higher ECG concentration can produce better regenerated cartilage has not been investigated. In addition, some important questions, including how to precisely control the shape after injection and achieve stable regenerated cartilage, still need to be studied.

## 5 Conclusion

This study establishes and demonstrates a new concept for cartilage regeneration on the basis of an injectable platform of a ECG-GelMA construct. The ECG-GelMA construct has preferred gel characteristics, superior biocompatibility, enhanced cartilage formation capacity, and enhanced mitigatory immunologic reaction compared with a traditional Cell-GelMA construct. The results of this study provide a new strategy for injectable cartilage regeneration with the potential for clinical translation.

## Data Availability

The original contributions presented in the study are included in the article/Supplementary Material, further inquiries can be directed to the corresponding authors.
