# Supplementary figures and images for: An Injectable Platform of Engineered Cartilage Gel and Gelatin Methacrylate to Promote Cartilage Regeneration
Source: Front Bioeng Biotechnol. 2022 Apr 14;10:884036. doi: 10.3389/fbioe.2022.884036 (PMC9074996; doi:10.3389/fbioe.2022.884036)

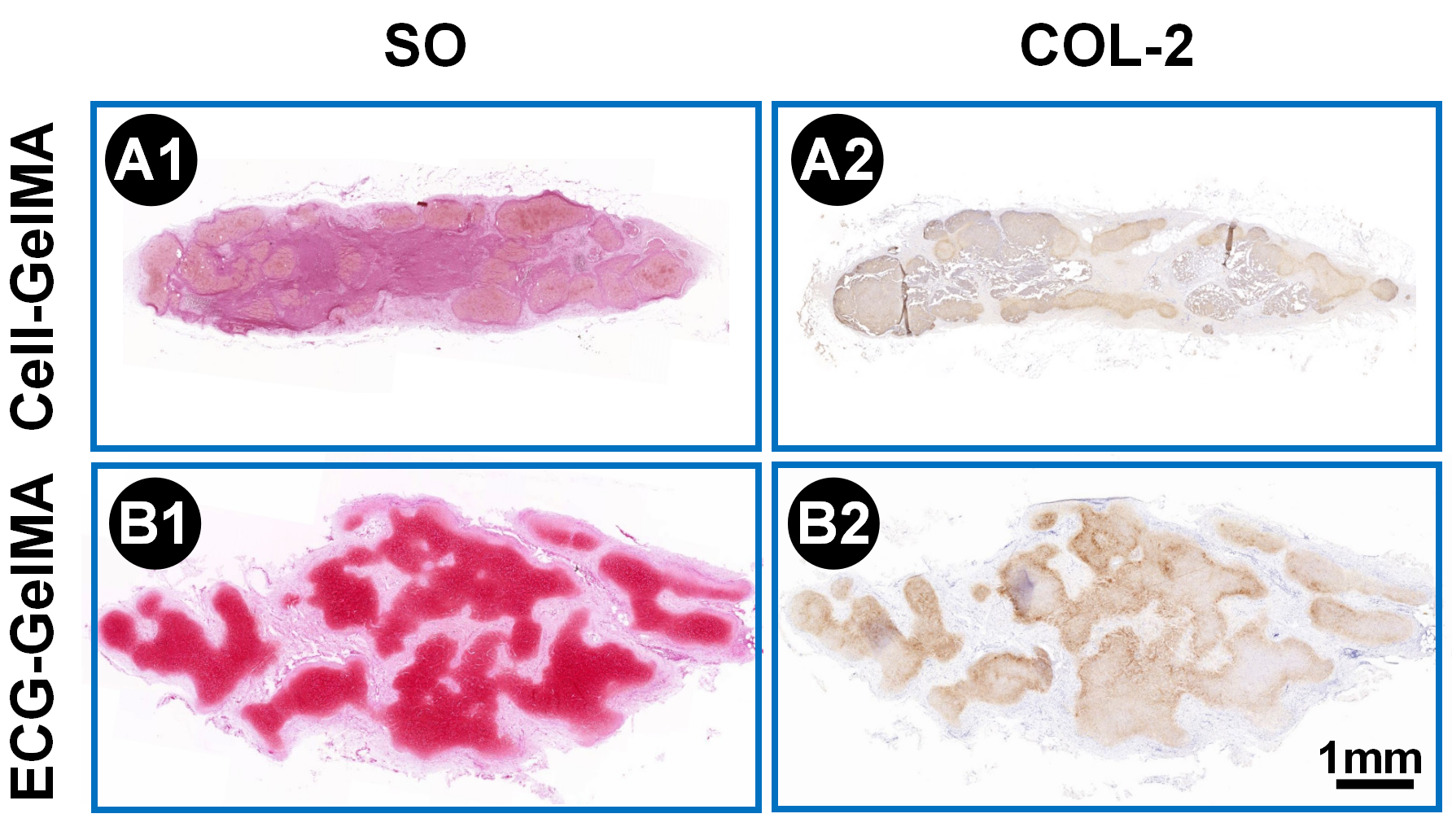

Supplement: Supplementary file 2 [file Image6.TIF]

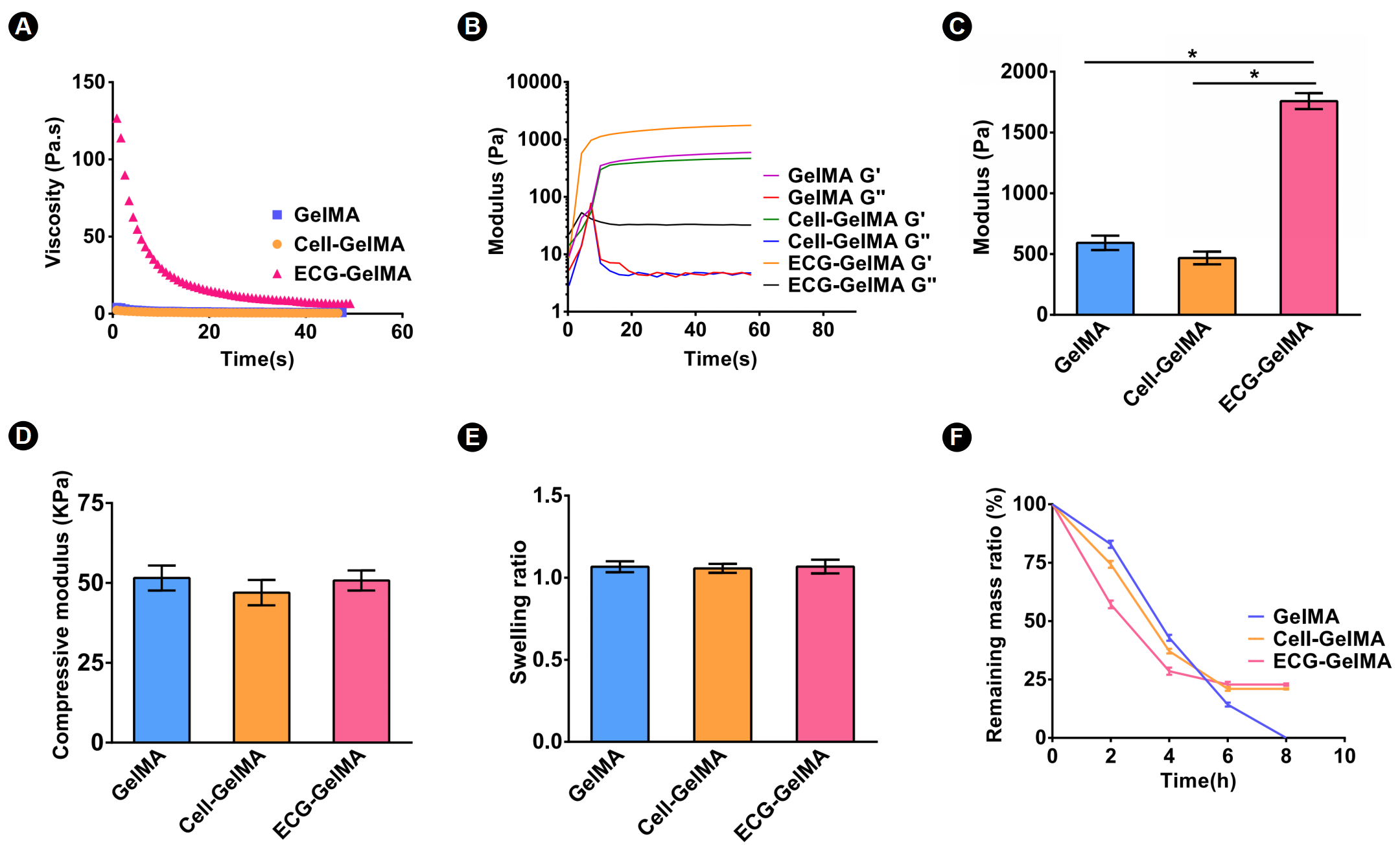

Supplement: Supplementary file 3 [file Image3.TIF]

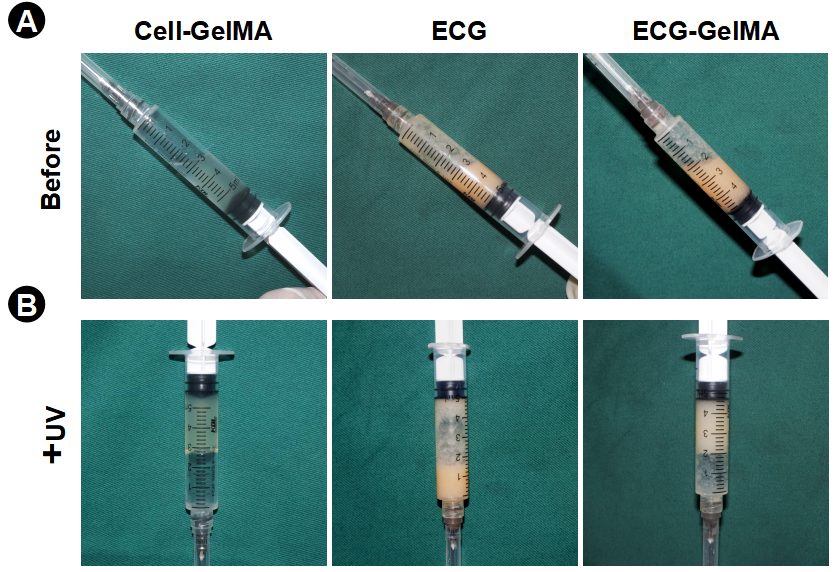

Supplement: Supplementary file 4 [file Image4.TIF]

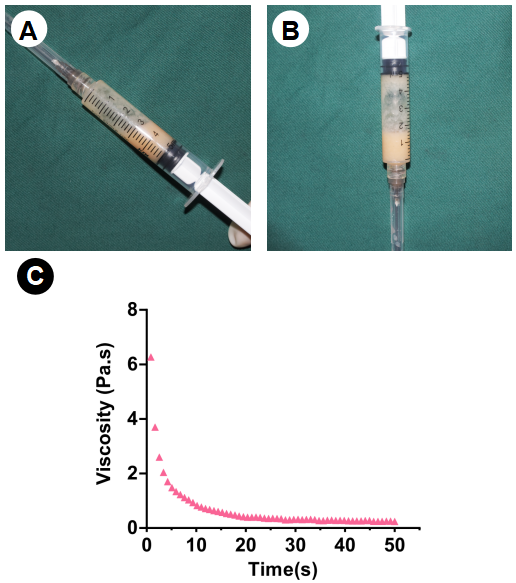

Supplement: Supplementary file 5 [file Image2.TIF]

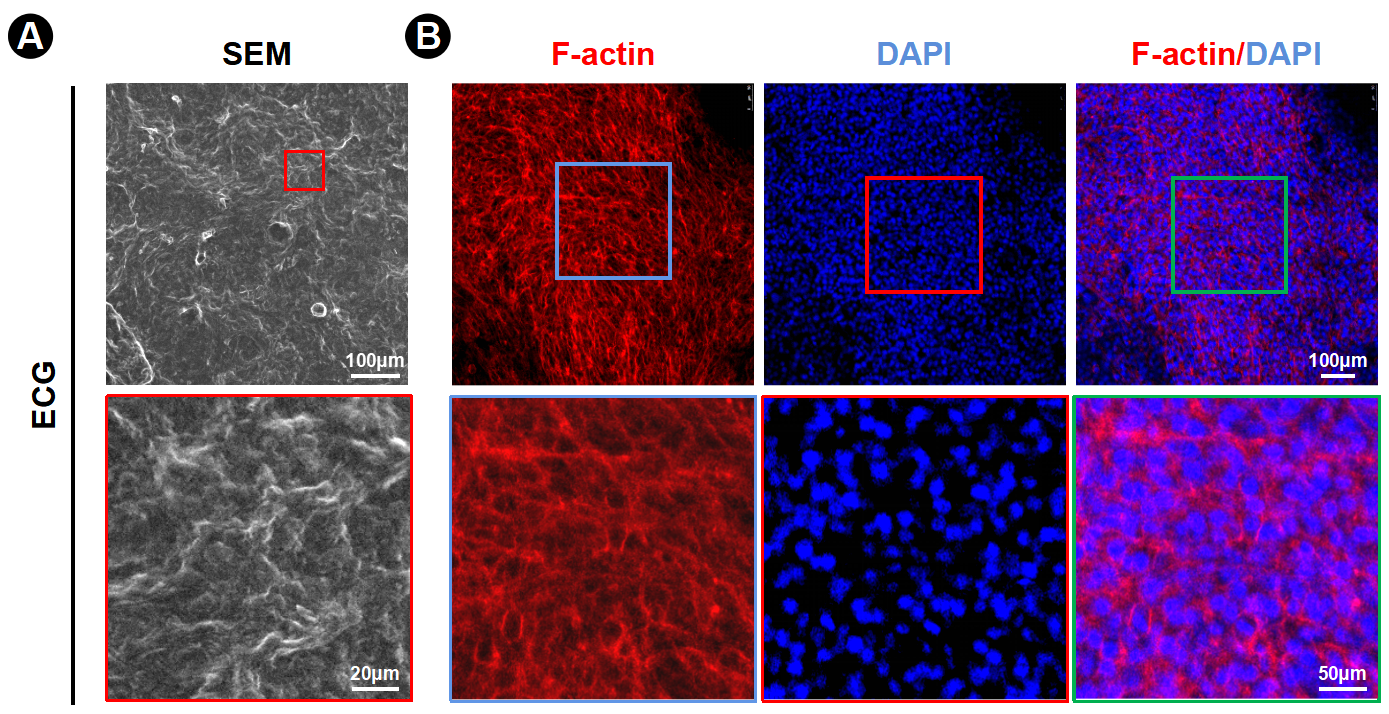

Supplement: Supplementary file 6 [file Image1.TIF]

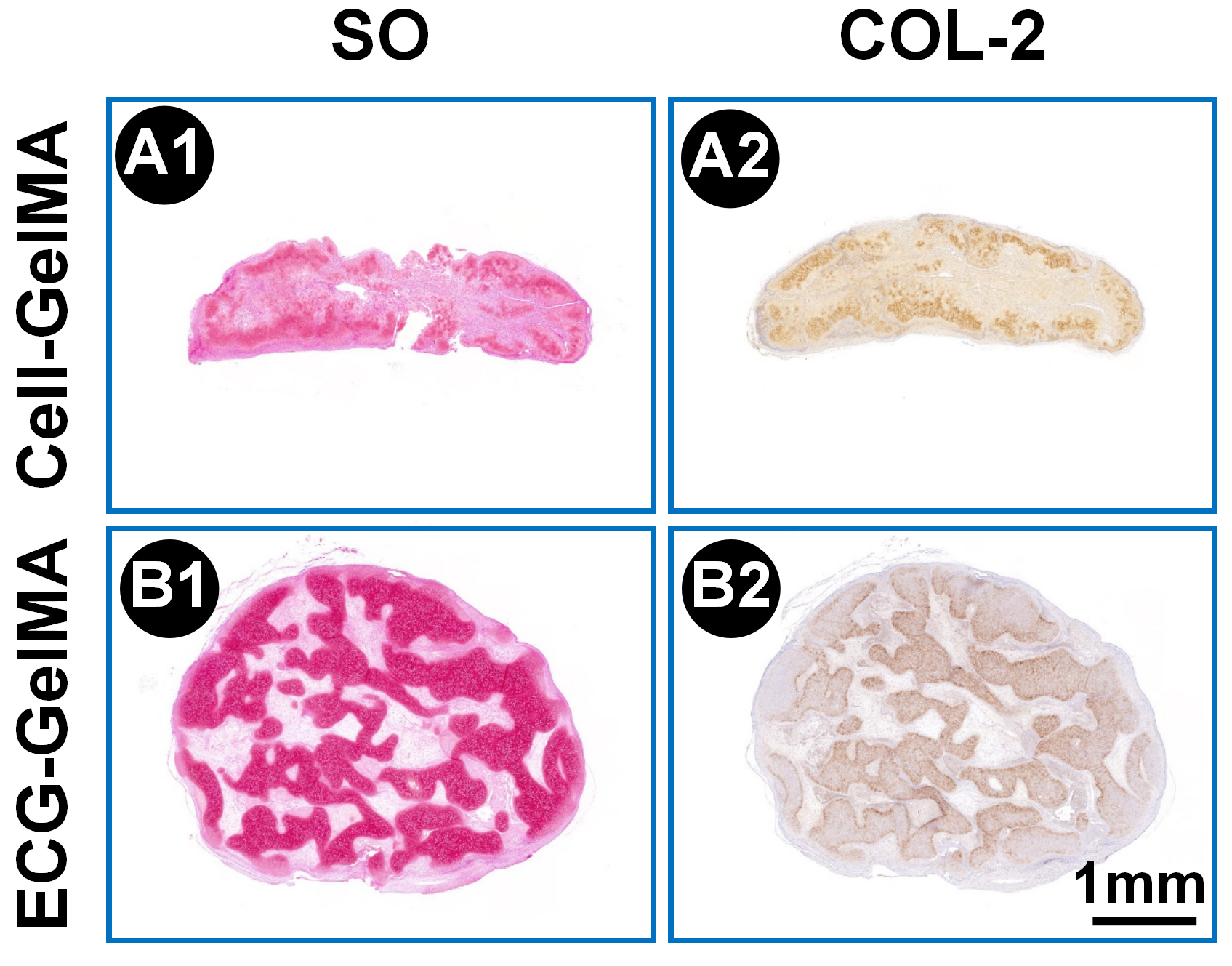

Supplement: Supplementary file 7 [file Image5.TIF]
