# Supplementary material for: An Injectable Platform of Engineered Cartilage Gel and Gelatin Methacrylate to Promote Cartilage Regeneration
Source: Front Bioeng Biotechnol. 2022 Apr 14;10:884036. doi: 10.3389/fbioe.2022.884036 (PMC9074996; doi:10.3389/fbioe.2022.884036)
Supplement: Supplementary file 10 [file DataSheet1.docx]

**Supporting Information**

**An Injectable Platform of Engineered Cartilage Gel and Gelatin Methacrylate to Promote Cartilage Regeneration**

Wei Xu^a,b,c,1^, Tao Wang^a,b,c,1^, Yahui Wang^b,1^, Xiaodi Wu^a,b,c^, Yujie Chen^d^, Daiying Song^a,b,c^, Zheng Ci^a,b,c^, Yilin Cao^b,c^, Yujie Hua^a,b,c,*^, Guangdong Zhou^a,b,c,*^, Yu Liu^a,b,c,*^

^a^ Research Institute of Plastic Surgery, Wei Fang Medical College, Weifang 261000, China

^b^ National Tissue Engineering Center of China, Shanghai 201100, China

^c^ Shanghai Key Laboratory of Tissue Engineering, Department of Plastic and Reconstructive Surgery, Shanghai 9th People’s Hospital, Shanghai Stem Cell Institute, Shanghai Jiao Tong University School of Medicine, Shanghai 200001, China

^d^ Shanghai Engineering Research Center of Nano-Biomaterials and Regenerative Medicine, College of Chemistry, Chemical Engineering and Biotechnology, Donghua University, Songjiang, Shanghai 201600, China

^1^ These authors are equal contributors to this work.

* Corresponding authors: Dr. Yujie Hua: [hyj137@shsmu.edu.cn](mailto:hyj137@shsmu.edu.cn); Dr. Guangdong Zhou, Email: [guangdongzhou@126.com;](mailto:guangdongzhou@126.com;) Dr. Yu Liu, Email: [yuliu1211@163.com](mailto:yuliu1211@163.com).


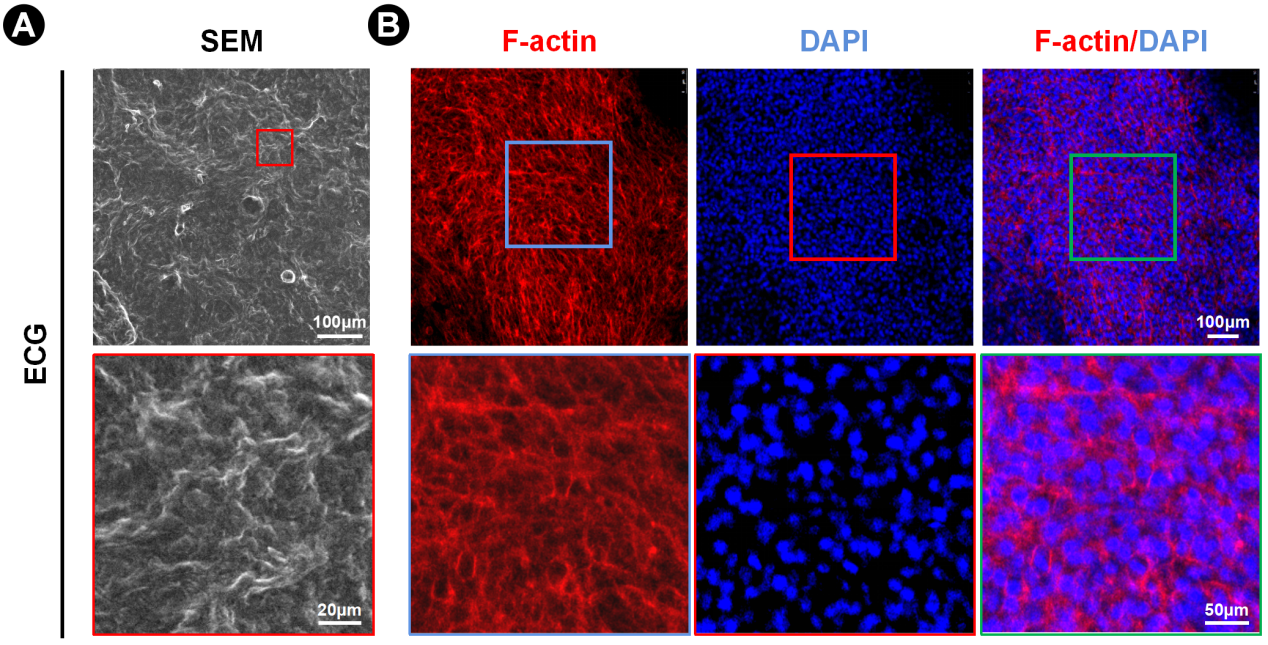


**Supporting figure 1.** SEM images of ECG after cultured for 5 days (A). Phalloidin staining images in ECG (B).


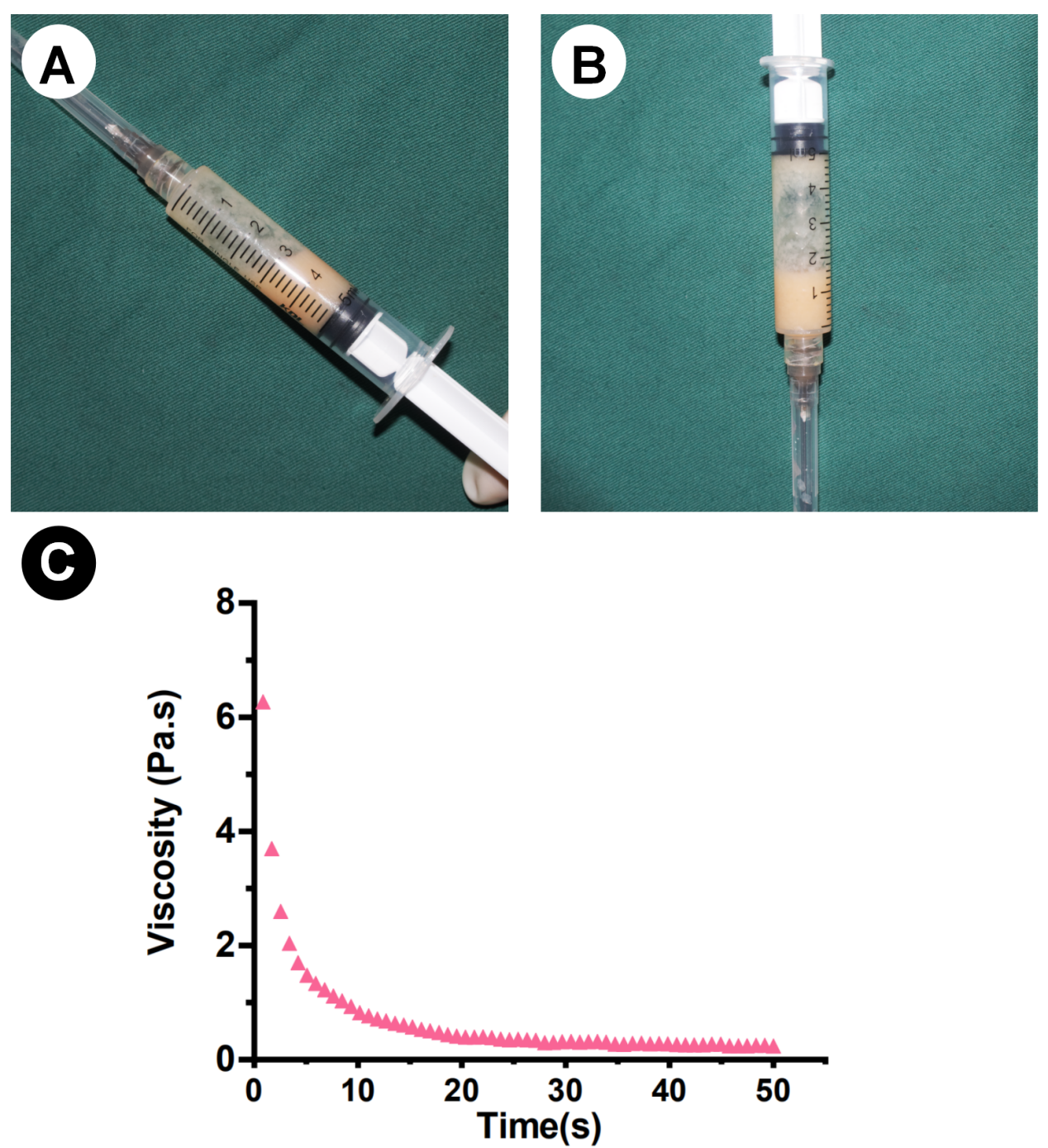


**Supporting figure 2.** Gel properties of ECG (A and B). Viscosity curve of ECG (C).


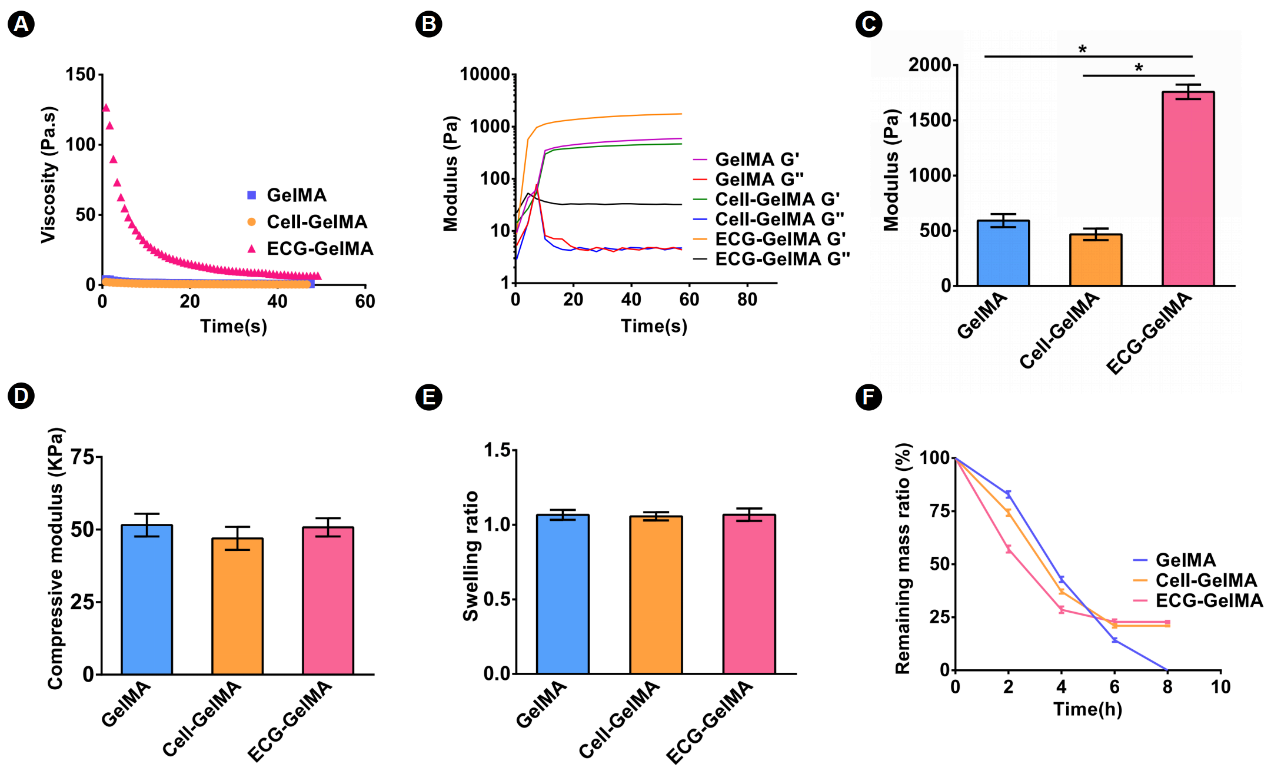


**Supporting figure 3.** Viscosity evaluation of Cell-GelMA, GelMA and ECG-GelMA (A), Time-sweep rheological curve (B), Storage moduli (C), compressive modulus (D), swelling ratio (Volume swelling ratio) (E), and degradation rate (F) in Cell-GelMA, GelMA and ECG-GelMA. **P* < 0.05.


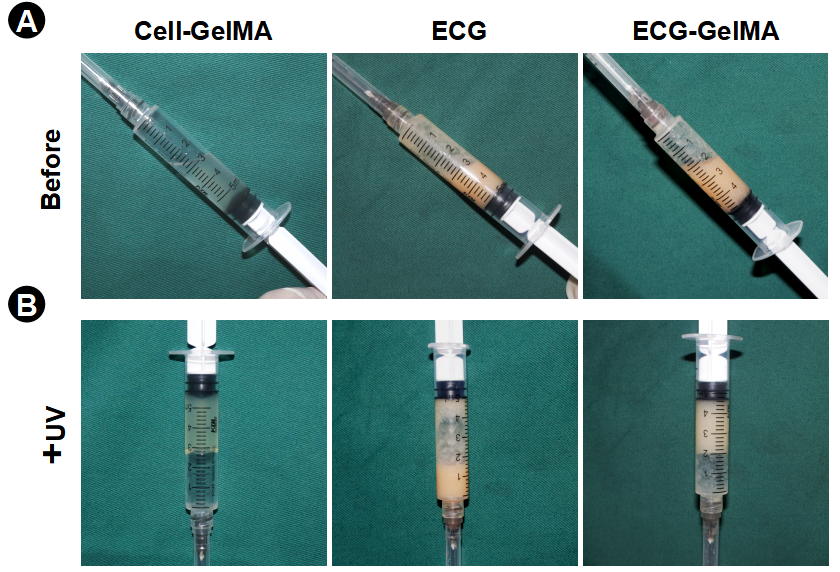


**Supporting figure 4.** Gel evaluation of Cell-GelMA, ECG, and ECG-GelMA before and after UV irradiation


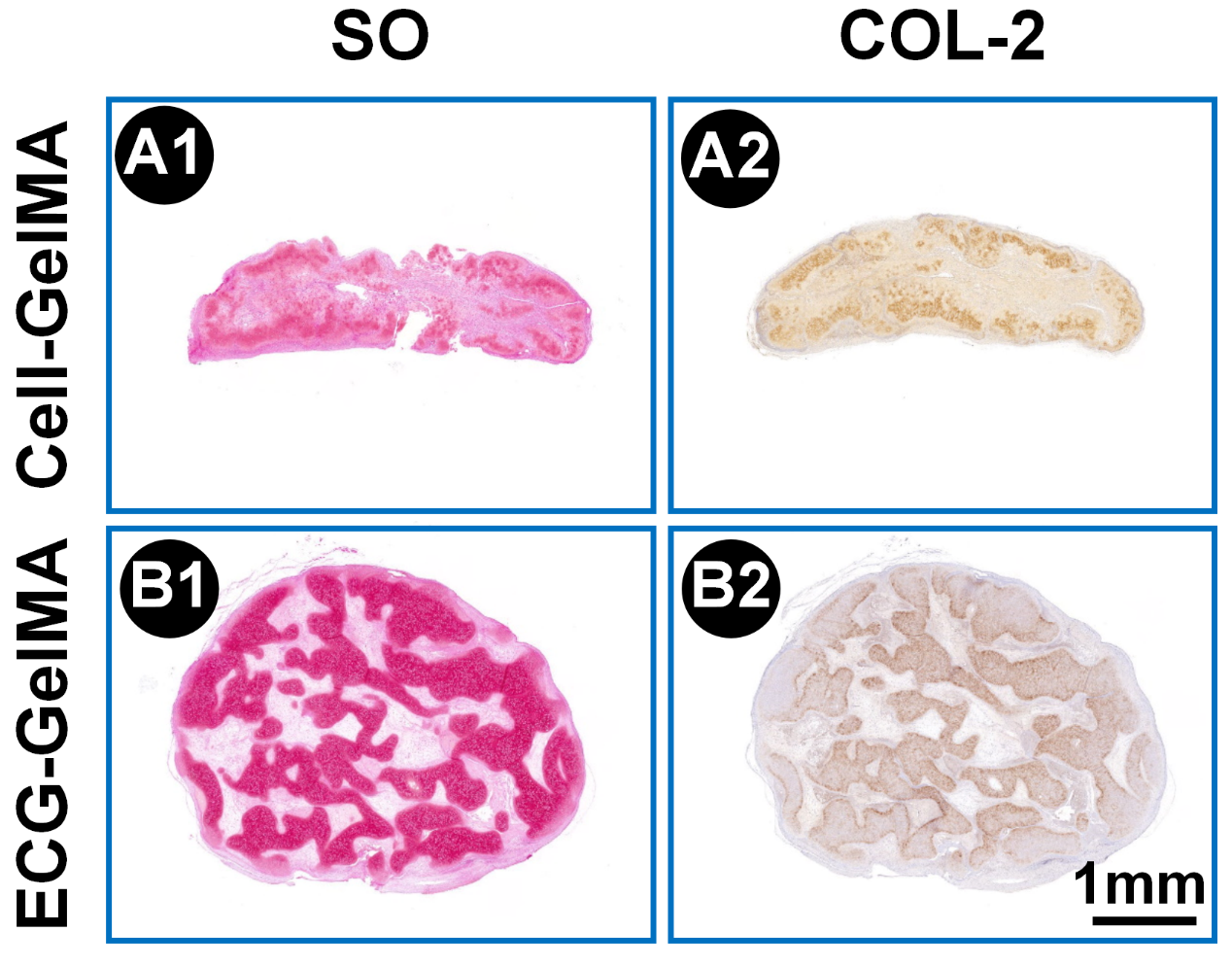


**Supporting figure 5.** The panorama SO (A1 and B1), and immunohistochemical COL-2 (A2 and B2) staining of Cell-GelMA and ECG-GelMA after 8 weeks implantation in nude mice.


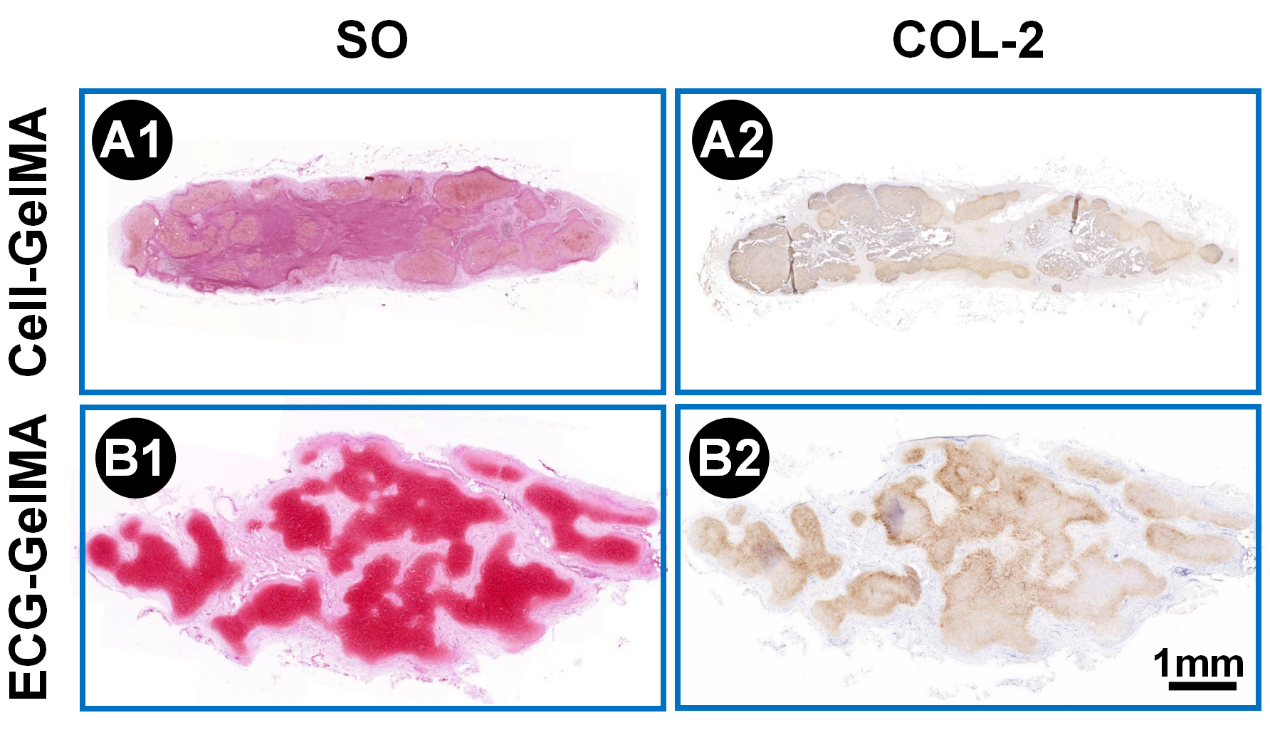


**Supporting figure 6.** The panorama SO (A1 and B1), and immunohistochemical COL-2 (A2 and B2) staining of Cell-GelMA and ECG-GelMA after 12 weeks implantation in goat.
